# Emotional Eating among College Students in Israel: A Study during Times of War

**DOI:** 10.3390/foods13091347

**Published:** 2024-04-27

**Authors:** Nourit Houminer Klepar, Nadav Davidovitch, Keren Dopelt

**Affiliations:** 1 Department of Public Health, Ashkelon Academic College, Ashkelon 78211, Israel; 2 Department of Health Policy and Management, Guilford Glazer Faculty of Business and Management and Faculty of Health Sciences, Ben-Gurion University of the Negev, Beer Sheva 84105, Israel

**Keywords:** emotional eating, stress, college students, times of war, Israel

## Abstract

Emotional eating, the act of consuming food to cope with negative emotions rather than responding to hunger cues, can lead to overeating in an attempt to regulate and alleviate these emotions. This study aimed to assess emotional eating among college students in Israel, specifically during times of war, which present unique and heightened stressors that accumulate on top. A total of 575 participants from the Ashkelon Academic College completed an online questionnaire examining background information, stress levels, and emotional eating symptoms. Our findings indicate that factors, such as being female, not having children, younger age, lower body satisfaction, higher BMI, and increased stress, are predictors of heightened emotional eating. These results highlight risk factors predisposing college students to engage in emotional eating. Developing targeted interventions, particularly campus-based programs to address emotional eating by promoting healthy coping strategies, a positive body image, and stress management skills is needed. In addition, raising awareness concerning emotional eating risks during challenging life transitions and distressing situations is necessary. The college leadership, led by the departments of Nutrition, Psychology, and Public Health, in collaboration with stakeholders in the Israeli Ministry of Health, must consider the mental effects of war on students and their involvement in emotional eating.

## 1. Introduction

Emotional eating (EE) is defined as consuming food to cope with emotions, such as stress, sadness, loneliness, or boredom, rather than responding to physiological hunger cues [1]. This may lead to overeating in an attempt to regulate and alleviate these negative emotions [2]. Individuals experiencing EE usually opt for convenient high-calorie foods, such as fast foods and sugary snacks, with low nutritional value [3,4]. Consumption of sweet and high-fat foods triggers the release of neurotransmitters like opioids and dopamine in the brain, linked with sensations of pleasure and reward, ultimately leading to mood enhancement and stress reduction [5]. Therefore, these food choices serve as a coping or healing strategy for emotional eaters seeking to manage their emotional well-being [6]. Nonetheless, consumption of these foods over time may lead to weight gain and obesity [7,8,9] and, consequently, to overweight-related diseases such as type 2 diabetes and cardiovascular diseases [10]. 

EE is increasingly recognized as a prevalent eating behavior rather than a distinct eating disorder. Therefore, there is a lack of standardized diagnostic criteria [11]. EE can be associated with different factors, such as coping with stress [12], higher weight status [13], obesity [14], negative body image [15], increased social media usage [16,17,18], gender [19,20,21], and age [17,22]. The multifaceted nature of EE and its associated factors requires further investigation to deepen our understanding and inform effective interventions. College students represent a particularly vulnerable group concerning EE due to various lifestyle changes accompanying the transition to college or university. This includes shifts in meal structures, increased access to diverse food options, and the necessity for independent dietary decision making, potentially diverging from the typically healthier, home-cooked meals often provided in home settings, making students more susceptible to EE and weight gain [11,23,24]. Moreover, academic stress, usually more pronounced among female students [25], and the pressure to succeed, including post-graduation plans, and the accompanied financial worries [25], may affect students’ food choices, often manifesting in reduced consumption of fruits and vegetables and the selection of unhealthy comforting foods and sugary beverages [26].

A survey involving 967 college students revealed that, on average, students exhibited moderate EE behavior, with approximately 10% showing signs of food addiction [27]. Another study, including 317 female college students, found that over 31% of them experienced a binge eating disorder [28]. In studies conducted in Hunan, China, where 1301 college students were included, and in Spain, with 584 university students, 53% and 39% reported high levels of EE, respectively [29,30]. In Israel, approximately 23% of adolescents, mostly girls, were found to have pathology related to eating, implying a risk of developing an eating disorder [31].

This research was carried out among students at the Ashkelon Academic College, located in southern Israel. In 2024, approximately 4200 students studied at the college on the academic track, coinciding with a significant period of conflict in the region, considered a state of war. 

Located just 14 km from the conflict area, the city of Ashkelon endures frequent rocket fire, contributing to heightened depression, stress, and anxiety among its residents, who endured the loss of loved ones and were forced to evacuate their homes. Despite this research being conducted four months after the initial significant attack, Israeli citizens continue to face the ongoing threat of existential war.

Stress during wartime can alter eating behaviors and increase the risk of developing eating disorders later on [32]. In November 2023, an online survey conducted in Israel among 501 residents of a community that experienced conflict starting on 7 October revealed notable shifts in behavior compared to pre-war times. Respondents indicated a notable uptick in tobacco (56%) and alcohol (15%) usage, along with a decline in sleep quality (63%) and heightened distress levels. Additionally, over a third of participants reported experiencing changes in weight. Notably, distress levels were found to be particularly elevated among female respondents [33]. A recent quantitative investigation delved into the frequency of mental health disorders among civilians in Ukraine impacted by the continuing conflict. The study revealed troubling levels of trauma-related conditions, such as depression, anxiety, and post-traumatic stress disorder, within the surveyed populace [34].

Given these statistics, there is a critical need for a survey targeting EE among college students in Israel, as well as the factors associated with EE behavior. Notably, this is the first research in Israel to examine EE among college students, specifically during times of war, emphasizing the unique and heightened stressors faced by participants beyond the common stressors that college students experience. We hypothesize that women are more vulnerable to emotional eating compared to men and that there is a positive correlation between stress and emotional eating.

## 2. Materials and Methods

### 2.1. Procedure

The current study was a cross-sectional descriptive study. Approval for the study was obtained from the Ashkelon Academic College Ethics Committee (approval #47-2024). The survey questionnaire was conducted via Qualtrics (Qualtrics, Provo, UT, USA) and was emailed to all students through the college’s computing infrastructure. The study took place between 28 January 2024 and 25 February 2024. According to the software data, the average response time to the questionnaire was approximately 5 min. There were 767 entries for the survey, with 710 students initiating the questionnaire. A total of 575 students filled out the questionnaire. Consequently, the response rate was 75% of the total survey entries and 14% of the research population. The introduction page clarified the study’s objectives and guaranteed participant anonymity. By completing the questionnaire, students demonstrated their voluntary agreement and informed consent to partake in the study. Participants were free to cease their responses at any point, and there was no requirement to answer particular questions.

### 2.2. Tools

We employed an anonymous, online, closed, self-completed questionnaire for data collection. The questionnaire was presented to ten students not affiliated with Ashkelon Academic College to assess the clarity of the questions. Feedback from this pilot test was used to revise three questions. Additionally, the questionnaire underwent content validation through evaluation by three experts: a public health researcher, a nutritionist, and a psychologist.

The questionnaire consisted of three parts:

Demographic and general information included the following: gender, age, marital status, religion, college department, height, weight, body satisfaction (on a scale of 1–5), estimation of the average time spent on social media per day (Instagram, Facebook, YouTube), residing in the conflict zone (yes/no).

War stress was assessed via three items: “Since the war began, how often have you felt (1) anxious about something unexpected that happened, (2) unable to control important aspects of life, (3) angry about things beyond their control?” Respondents were requested to indicate the frequency of each feeling using a Likert scale spanning from 1 (not at all) to 5 (often). War stress was calculated for each participant by summing and then averaging the 3-item responses, with a higher score indicative of higher levels of stress. Cronbach’s α = 0.74.

Emotional eating comprised five statements taken from Chesler et al. [35]. The statements present various methods of utilizing food to alleviate negative emotions. Participants were prompted to indicate the applicability of each method by responding “True” or “False” based on their experiences over the past few months. The variable EE was derived by tallying the “True” responses, with scores ranging from 0 to 5. A higher EE score suggests a greater inclination towards emotional eating. Cronbach’s α yielded a coefficient of 0.88, indicating high internal consistency.

### 2.3. Data Analysis

The data were analyzed using SPSS 29.01 (IBM, Armonk, NY, USA). Since the variables satisfied the criteria of normal distribution, differences between groups of gender, marital status, religion, and living in the conflict zone were examined using independent samples *t*-tests, and differences between groups of faculty and BMI were examined using one-way analyses of variance (ANOVA). Relationships between age, stress, time spent on social media, and EE were tested using Pearson correlations. Relationships between body satisfaction and EE were tested using Spearman correlation. A multiple linear regression model was used to predict the extent of EE. The model included variables that have been found to be linked with EE in the univariate analyses. The model incorporated variables, including gender, parental status (having children or not), age, body satisfaction, BMI, stress levels, and time spent on social media. Significance in reported *p*-values was determined through two-sided tests, where values below 0.05 were considered significant.

## 3. Results

### 3.1. Participant Characteristics

In total, 575 students participated in this study, of whom 72% were women, 52% were in relationships, and 27% had children. Most participants were Jewish (80%), and 42% lived in the conflict zone. More than half of the students studied in the Faculty of Social Sciences (57%), 25% in Health Sciences, and 18% in Computer Science and Management. The mean age of participants was 27.82 ± 8.87 years. The survey population resembles the college population in terms of gender, age, and faculty composition. Twenty-five percent of participants expressed dissatisfaction with their bodies, while one-third reported moderate satisfaction, leaving 41% categorized as either quite or very satisfied. The Body Mass Index (BMI) ranged from 15.2 to 42.3, with a mean of 24.4 ± 5.12. Over half of the participants fell within a normal weight range (56%), 8% were underweight, 21% were overweight, and 15% were classified as obese. The characteristics of the participants are summarized in Table 1.

### 3.2. Emotional Eating

Table 2 displays the distribution of responses pertaining to EE, as addressed in the questionnaire.

To assess the EE variable, the “true” responses were tallied for each participant. The EE average was 2.40 ± 1.04. About 37% were categorized as emotional eaters (scores 4–5 in the EE variable).

### 3.3. War Stress

The distribution of responses to the questionnaire, which specifically evaluates stress levels associated with the onset of the war, is presented in Table 3, after combining categories as follows: answers 1 and 2 were incorporated into the category “seldom”, while answer 3 was classified as “sometimes”, and answers 4 and 5 were integrated into the category “often”.

The stress level was assessed by computing the mean for each student, resulting in a value of 3.20 ± 0.95.

### 3.4. Relationships between Study Variables and EE

Table 4 presents the differences between various groups concerning EE. The differences were examined using independent samples t-tests or one-way ANOVA according to the independent variable scale. It also presents the relationship between age, body satisfaction, stress, and time spent on social media and EE. Relationships between variables were tested using Pearson and Spearman correlations according to the variables scale.

This study identified significant differences between student demographics and EE. Female students reported higher levels of EE compared to males. Additionally, students without children reported greater EE than students with children. Furthermore, analysis of BMI categories revealed a decreasing trend in EE overweight students, who reported the highest levels of EE, followed by obese and normal-weight students, respectively. Underweight students exhibited the lowest levels of EE.

Moreover, statistically significant, moderate negative correlations were observed between age and body satisfaction with EE (r_p_ = −0.19, *p* < 0.001; r_s_ = −0.20, *p* < 0.001), indicating that younger students and those with lower body satisfaction reported higher levels of emotional eating. Stress and daily social media use both exhibited significant, moderate, and positive correlations with EE (r_p_ = 0.15, *p* < 0.001; r_p_ = 0.20, *p* < 0.001, respectively). This implies that students experiencing higher stress and spending more time on social media reported engaging in EE more frequently.

### 3.5. Regression Model to Predict EE

A linear regression model was employed to identify significant predictors of EE in students. The model incorporated variables including gender, parental status (having children or not), age, body satisfaction, BMI, stress levels, and time spent on social media. The results are presented in Table 5. The analysis revealed that several factors were significantly associated with higher levels of EE (*p* < 0.001), including being female, not having children, younger age, lower body satisfaction, higher BMI, and experiencing greater stress, which were all found to be predictors of higher EE. The explained variance of the model was 14% (*p* < 0.001).

## 4. Discussion

This study investigated EE among college students at Ashkelon Academic College in Israel during a time of war. Understanding EE behaviors in this context is crucial, given the unique stressors faced by individuals in general and, in particular, among students residing in conflict zones.

Consistent with the existing literature, our findings revealed that various factors, including gender, parental status, age, body satisfaction, BMI, and stress levels, influence EE. Notably, female students reported significantly higher levels of EE compared to males, aligning with previous research suggesting gender differences in emotional eating behaviors [20,36,37]. This gender difference might be attributable to different coping strategies between men and women. Women are often more emotionally expressive and, therefore, use food to comfort them [38], whereas men often suppress emotions and may seek alternative coping strategies [39].

Additionally, the results demonstrated a negative relationship between emotional eating and age, suggesting that younger students may be more vulnerable to using food to regulate emotions. This aligns with previous work by Samuel and Cohen [22], who demonstrated that emotional eating, even though it is present among adults, gradually decreases with age. This could be due to better emotional regulation among adults or better coping mechanisms acquired through adulthood [40].

Furthermore, our results indicated that students without children reported greater EE than those with children. The findings suggest that parental responsibilities may have a protective effect against emotional eating behaviors, possibly due to the time-consuming caregiving responsibilities, which could limit the time available for emotional eating. Moreover, students with children who follow positive feeding styles, providing high structure and control, may have more structured meal routines and priorities that reduce emotional influences on eating behavior [41].

Additionally, body dissatisfaction and BMI emerged as significant predictors of EE, with overweight and obese students exhibiting higher levels of EE compared to those with normal weight or underweight status, supporting previous evidence linking negative body image and overweight/obesity status with emotional eating tendencies [42]. Higher BMI could arise either from overeating provoked by EE or, on the contrary, EE may result as a coping mechanism for distress related to being overweight. It has been shown that overweight individuals consume more food with negative emotions, while underweight individuals, interestingly, tend to consume more food with positive emotions [13]. In sum, Individuals dissatisfied with their bodies may be more inclined to use food for emotional regulation. Emotional eaters consume more sweet and fatty foods during times of stress [43], which can compromise their health in the long run if they experience stress chronically.

Likewise, greater time spent on social media correlated positively with EE, in accordance with previous findings [16,17,18]. The pervasive influence of social media on body image concerns and societal pressures around physical appearance may contribute to disordered eating behaviors like EE.

Although various studies assessed the effect of stress on emotional eating and food choices, minimal studies assessed emotional eating during times of war. Jayne et al. [44] studied emotional eating among soldiers and found that EE might mediate the relationship between stress and body weight, indicating that stress negatively impacts force readiness. In a similar manner, a study conducted during the COVID-19 pandemic serves as an illustrative example of research examining emotional eating behaviors amidst a major stressor or disaster. The fear of infection, uncertainty, and prolonged periods of social distancing and quarantine contributed to heightened levels of stress, which consequently increased emotional eating patterns, such as consumption of comfort foods to lessen negative emotions [45].

Overall, our study contributes to the growing body of literature on EE among college students. The unique context of this study, conducted amidst an active war situation, accentuates the potential exacerbating influence of severe environmental stressors on emotional eating patterns. Further research is warranted to explore the longitudinal trajectories of EE and its implications for mental and physical health outcomes among college students in conflict-affected regions, alongside the influence of the individual experiences of the participants regarding the war.

### Study Limitations

The current research was restricted to students from a solitary college, potentially constraining the generalizability of these findings to students across the country. In addition, 72% were women, which may bias the research results. However, it represents the percentage of women in the college. Furthermore, the survey was conducted four months after 7 October, which marked the start of the war, and participants returned to their normal routines and studies. It can be assumed that if we had administered the survey shortly after 7 October, the stress levels would have been much higher. Moreover, this study lacks an individualized approach; i.e., the individual experiences of the participants regarding the war were not examined. Nevertheless, this research provides a novel investigation into EE within the unique circumstances encountered by Israeli students and highlights the necessity for continued exploration of this complex eating behavior across diverse populations and settings.

## 5. Conclusions

On a theoretical level, we proposed a model of variables related to emotional eating among students in general and specifically during times of war. The results highlight demographic, psychological, and environmental risk factors predisposing college students to engage in EE. Developing targeted interventions, particularly campus-based programs to address EE by promoting healthy coping strategies, positive body image, and stress management skills, as well as raising awareness about EE risks during challenging life transitions and distressing situations, may also be beneficial.

The college leadership, led by the departments of Nutrition, Psychology, and Public Health, in collaboration with stakeholders in the Israeli Ministry of Health, must consider the mental effects of war on students and their involvement in emotional eating. Emotional eating can lead to eating disorders, highlighting the urgency of addressing this phenomenon. Supporting groups on campus can assist students in coping with the repercussions of war on their health behaviors, particularly regarding emotional eating.

## Figures and Tables

**Table 1:** The characteristics of study participants.

Characteristics	*n*	%
Gender:		
Male	159	28
Female	416	72
In relationship	296	52
Have children	155	27
Jewish	460	80
Faculty:		
Social Sciences	330	57
Health Sciences	142	25
Computers & Management	103	18
Living in the conflict zone	242	42
Time spent on social media per day:		
Doesn’t have any social media account	64	11
Doesn’t use social media	13	2
Up to one hour	78	14
Between 1–2 h	121	21
Approximately 2–3 h	144	25
More than 3 h	155	27
Body satisfaction:		
Weakly (answers 1 + 2)	143	25
Moderately (answer 3)	195	34
Strongly (answers 4 + 5)	237	41
BMI:		
Underweight (BMI < 18.5)	43	8
Normal (18–24.9)	316	56
Overweight (25–29.9)	121	21
Obesity (BMI > 30)	86	15

**Table 2:** The distribution of answers to the EE questionnaire.

Statement	True (%)
Eating helps me get through tough times	55
When I have problems, food is like my best friend	38
Food comforts me when I am angry	46
When I am sad, food consoles me	55
Food calms me when I am anxious	47

**Table 3:** The distribution of answers to the questionnaire focused on stress.

Statement	Seldom (%)	Sometimes (%)	Often (%)	Mean ± SD *
Anxious about something unexpected that happened	17	32	51	3.49 ± 1.16
Unable to control important aspects of life	34	34	32	2.92 ± 1.18
Angry about things that were beyond my control	26	35	39	3.19 ± 1.13

* SD = Standard deviation.

**Table 4:** Relationships between study variables and EE.

Variable	Groups	Mean ± SD *	F/t/r	*p*
Gender	Male	2.00 ± 1.09	_t(573)_ = 2.94	0.003
	Female	2.56 ± 1.01		
In relationship	Yes	2.28 ± 1.05	t_(573)_ = 1.47	0.074
	No	2.53 ± 1.03		
Have children	Yes	2.17 ± 1.01	t_(573)_ = 1.68	0.047
	No	2.49 ± 1.05		
Religion	Jewish	2.38 ± 1.03	t_(573)_ = 0.59	0.555
	Not Jewish	2.50 ± 1.10		
Faculty	Social Sciences	2.51 ± 1.04	F_(574)_ = 1.54	0.215
	Health Sciences	2.37 ± 1.04		
	Computers & Management	2.11 ± 1.05		
Living in the conflict zone	Yes	2.50 ± 1.04	t_(568)_ = 0.85	0.394
	No	2.35 ± 1.06		
BMI	Underweight	1.93 ± 0.96	F_(565)_ = 4.39	0.005
	Normal weight	2.20 ± 1.01		
	Overweight	2.66 ± 1.03		
	Obesity	2.93 ± 1.09		
Age			r_p_ = −0.19	<0.001
Body satisfaction			r_s_ = −0.20	<0.001
Stress			r_p_ = 0.15	<0.001
Time spent on social media			r_p_ = 0.20	<0.001

* SD = Standard deviation.

**Table 5:** Linear regression model results for predicting EE.

Variable	B	β	*p*
Gender (0—male, 1—female)	0.48	0.11	0.011
Having children (0—no, 1—yes)	0.61	0.13	0.020
Age	−0.07	−0.31	<0.001
Body satisfaction	−0.20	−0.11	0.015
BMI	0.08	0.19	<0.001
Stress	0.25	0.12	0.006
Time spent on social media	0.11	0.08	0.076
Adjusted R Square	0.14, *p* < 0.001		
F	14.23, *p* < 0.001		
N	558		

## Data Availability

The original contributions presented in the study are included in the article, further inquiries can be directed to the corresponding author.
